# The neglected pathogen: case reports of severe lower respiratory tract infection by human coronavirus 229E

**DOI:** 10.1099/acmi.0.000311

**Published:** 2022-02-10

**Authors:** Diptanu Paul, Akshita Gupta, Vikram Bhatia, Ekta Gupta

**Affiliations:** ^1^​ Department of Clinical Virology, Institute of Liver and Biliary Sciences (ILBS), New Delhi, India; ^2^​ Department of Hepatology, Institute of Liver and Biliary Sciences (ILBS), New Delhi, India

**Keywords:** coronavirus 229E, case report, lower respiratory tract infection, neglected

## Abstract

As the severe acute respiratory syndrome coronavirus 2 (SARS-CoV-2) pandemic continues, other previously ignored viruses must be taken into account as causes of severe acute respiratory distress, influenza-like illness and pneumonia. In this article, we report two cases of pneumonia in chronic liver disease patients where human coronavirus (HCoV) 229E was identified as the only infecting pathogen. Both the patients presented with fever, cough and respiratory distress, along with radiological findings suggestive of pneumonia. Multiplex real-time PCR for various respiratory viruses (FilmArray Respiratory Panel 2 *plus*) detected HCoV-229E in both cases. Both cases were managed with prophylactic antibiotics, steroids and supplemental oxygen therapy, after which they recovered completely and were discharged.

## Introduction

Coronaviruses are enveloped, single-stranded RNA viruses containing the largest genome among all RNA viruses. They have characteristic club-like projections of spike proteins on their surface, giving them the crown-like appearance for which they have been named (*corona* in Latin means crown) [1]. Aside from three deadly coronavirus species – severe acute respiratory syndrome (SARS), middle east respiratory syndrome (MERS) and severe acute respiratory syndrome coronavirus 2 (SARS-CoV-2) – four more known species of coronaviruses cause mild to moderate respiratory tract infections. These include human coronavirus (HCoV) 229E, HCoV OC43, HCoV NL63 and HCoV HKU1. Among these different known human coronaviruses, HCoV-229E was identified earliest in 1966 and is mainly known to cause the common cold in adults [2, 3]. However, in children, elderly and immunocompromised patients it can cause severe lower respiratory symptoms such as pneumonia and bronchiolitis [4, 5]. In otherwise healthy patients, these less severe viral infections can lead to secondary bacterial infections or other viral co-infections by disrupting the normal respiratory epithelial lining and lowering immunity, which further worsens the situation [6]. Moreover, delay in presentation due to overlapping signs and symptoms and self-medication of antibiotics not only delays early diagnosis and proper management but also adds to the burden of drug resistance.

With the advent of advancements in molecular diagnostics and the availability of combined multiple pathogen detection assays, viral pathogen aetiology identification has become easier. Viral aetiology should be identified in any case of pneumonia for proper medical management wherever available and to prevent unnecessary use of antibiotics.

In this era of SARS-CoV-2, the global focus has been on a single respiratory virus. However, it is important to re-evaluate the role of non-pandemic HCoV strains in severe respiratory illnesses. In this report, we present two cases of HCoV-229E infection causing respiratory distress and pneumonia among chronic liver disease patients.

## Case report

### Case 1

**Fig. 1.**
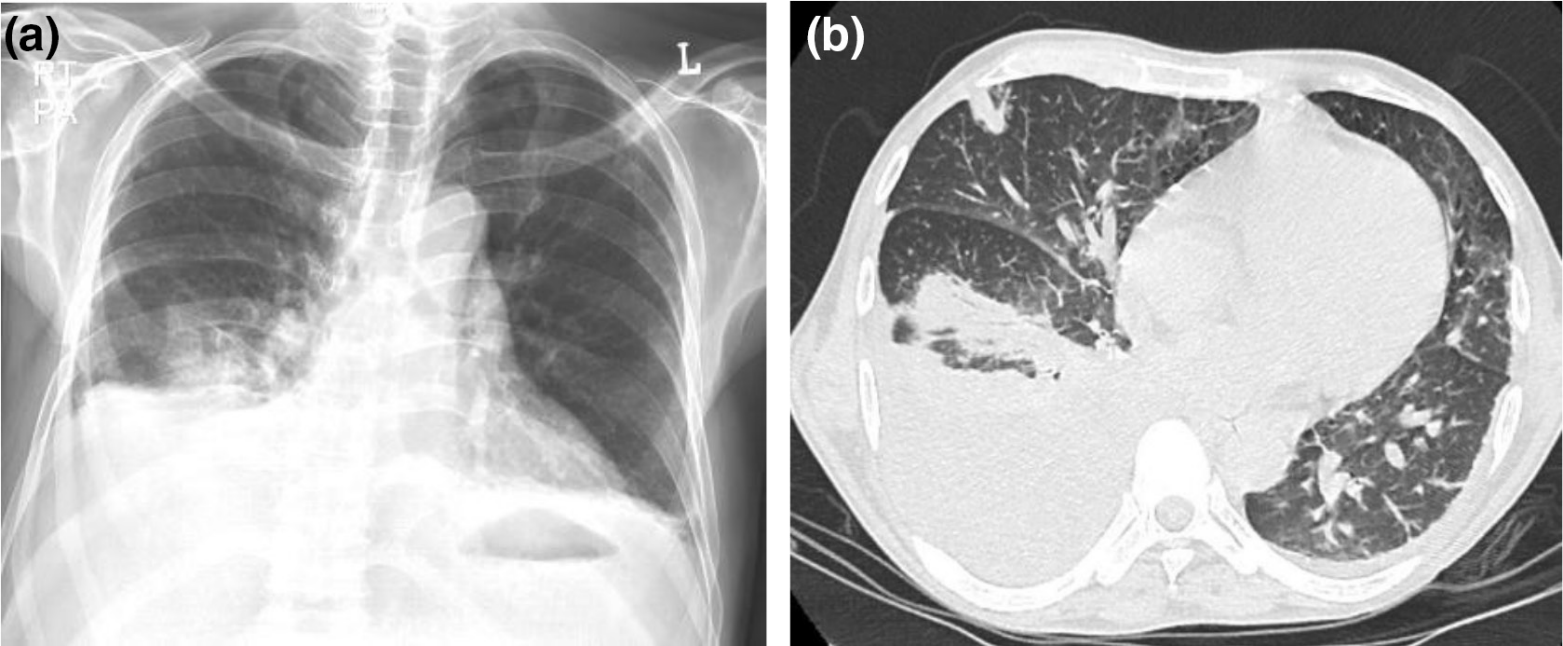
(**a**) Chest X-ray (PA view) showing pleural effusion and (**b**) HRCT of the chest (axial view) for case 1 depicting multiple nodules.

A 50–60-year-old patient presented to the emergency department with gradual onset of cough and mucoid expectoration, headache and fever up to 101 ° F for 3 days. He had decompensated alcohol-related cirrhosis of the liver. He was also a chronic smoker with no other medical co-morbidities. He was not on steroids or any immunosuppressant medications. He had not taken any over-the-counter antibiotics or any other medications except for paracetamol 500 mg for symptomatic relief and steam inhalation prior to hospital admission. On examination, there were diffuse rales present over the bilateral lung fields. His basic laboratory parameters are described in Table S1. Chest X-ray revealed right pleural effusion. High-resolution computed tomography (HRCT) of the chest showed multiple discrete rounded nodules, with areas of confluence in bilateral upper and right middle lobes and moderate right pleural effusion (Fig. 1). He was started on azithromycin and levofloxacin prophylactically.

The patient was repeatedly tested for SARS-CoV-2 on the first and second day of admission to the hospital by both rapid antigen test and real-time reverse transcriptase PCR (RT-rtPCR) targeting the E and RdRp genes due to the ongoing pandemic and heightened clinical suspicion. He was repeatedly negative. He was also negative for antibodies to SARS-CoV-2. The patient’s condition deteriorated further in the next 2 days with worsening dyspnoea, tachypnea, persistent fever (101 ° F), SpO_2_ 95–97 % and PaO_2_ 87.5 mmHg. Combined nasopharyngeal (N/P) and oral swabs were collected from the patient in viral transport medium (VTM) on the third day of admission and sent for identification of other viral causes of respiratory tract infections, which was performed using the BIOFIRE Film Array Respiratory Panel (RP) 2.0 *plus* (Biofire) (BioFire Diagnostics, LLC, Salt Lake City, UT, USA). The sample was positive for HCoV-229E. Simultaneously, a sputum sample was collected from the patient and was sent for bacterial and fungal cultures, which were negative for any bacterial and fungal pathogen. A repeat Biofire viral panel from both a combined NP/oral swab and a sputum sample after 2 days again showed a similar result. This was done to reconfirm the earlier findings, as it is unusual for HCoV-229E to be causing lower respiratory tract infection. The prophylactic antibiotic was immediately stopped after a negative bacterial report. The patient gradually improved with high-flow oxygen and standard medical management (Table S1). He did not need mechanical ventilation and was discharged after 7 days of hospitalization.

### Case 2

**Fig. 2.**
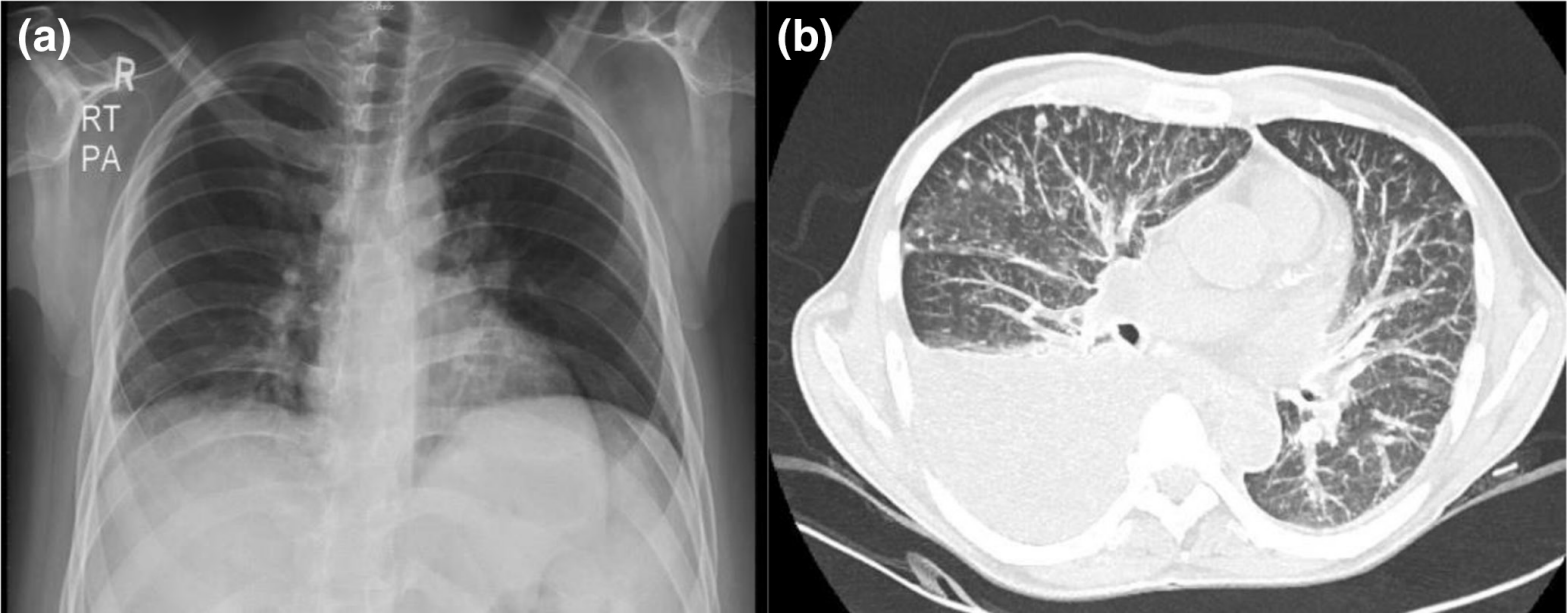
(**a**) Chest X-ray (PA view) showing pleural effusion and (**b**) HRCT of the chest (axial view) for case 2 showing ill-defined nodules, ground-glass opacity and septal thickening.

A 20–30-year-old patient presented with abrupt onset of fever up to 102 ° F for 4 days and gradual onset of breathlessness with productive cough for 2 days. He is a known case of alcoholic liver cirrhosis with jaundice, melaena and portal hypertension. He did not have any other comorbidities. There is no history of antibiotic or antiviral intake other than paracetamol 500 mg for fever prior to the hospital admission. On examination, there was a bronchial breath sound with diffuse rales present over the bilateral lung fields. The patient’s basic laboratory parameters are presented in Table S1. Chest X-ray showed mild right-sided pleural effusion. HRCT of the thorax showed multiple small ill-defined nodules in bilateral lung fields along with ground-glass opacity in both lung fields and septal thickening in bilateral lower lobes (Fig. 2). RTPCR and rapid antigen testing for SARS-COV-2 were negative on admission and were repeated twice over a period of 5–6 days, and these were also negative. The patient was also negative for SARS-CoV-2 antibodies.

Both combined NP/oral swabs in VTM for respiratory viral identification and sputum for bacterial and fungal identification were collected. Bacterial and fungal analysis was negative, while the respiratory viral panel detected HCoV-229E by Biofire. Repeat sputum and NP/OP swab tests on the Biofire viral panel resulted in HCoV-229E detection, which was done for reconfirmation of the earlier laboratory results days after the initial report. The patient’s general condition deteriorated, with persistent fever, dyspnoea, diffuse rales and altered ABG analysis – pH 7.447, PaCO_2_ 32.3 mmHg, PaO_2_ 50.7 and P/F 241.4 at FiO_2_ 21 %. He was treated with high-flow oxygen, corticosteroids (Table S1). The antibiotics levofloxacin and azithromycin, which were administered prophylactically, were stopped after a negative bacterial report. The status of the patient remained stable. Gradually over the next 4 days the patient recovered completely and was discharged.

## Discussion

Coronavirus HCoV-229E is known to be one of the common causes of upper respiratory tract infections (common cold) [7], but lower respiratory tract infections such as pneumonia are rarely reported in immunocompetent adults [8]. The prevalence of human coronaviruses in cases of upper respiratory tract infections is reported to be 10–30 %, but the individual prevalence of HCoV-229E is not exactly known [9]. HCoV-229E has an incubation period of 2–5 days with symptoms that are largely indistinguishable from those of other viral agents, including nasal discharge, sneezing, sore throat, cough, fever, headache and malaise [10]. This clinical picture can be different in high-risk groups such as infants, the elderly and immunocompromised patients, where lower respiratory tract involvement can lead to significant lung infections [4, 11, 12]. Chronic liver disease (CLD) is an important risk factor in terms of respiratory viral infections and has been known to involve severe clinical courses in comparison to healthy patients. In a previous study from our laboratory, we found a prevalence of 2.2 % of human coronaviruses in pneumonia patients with CLD [13].

Both of the cases described in this study showed similar clinical presentation of upper respiratory tract infection and fever with a background of CLD, which gradually progressed to mild acute respiratory distress syndrome (ARDS) (P/F ratio 200–300), as per the Berlin definition of ARDS [14]. The laboratory diagnostic workup and radiological findings for both the patients were suggestive of pneumonia due to infective aetiology. In both the patients, combined NP/OP swabs were used as samples. Combined NP/OP swabs have been known to show excellent sensitivity and specificity in the detection of respiratory tract viruses over sputum [15]; hence they was used as a first-line sample in both cases. The Biofire test was repeated on sputum and repeated collection of NP/OP swabs on special request of the clinicians to reconfirm the laboratory findings. Bacterial and fungal cultures were negative and only HCoV-229E could be identified as the causative agent.

There are limited case reports of HCoV-229E in adult pneumonia patients [8, 16]. Again, interestingly, similar radiological findings of pleural effusion and ground-glass opacities have also been observed among patients suffering from the new SARS-CoV-2 infection [17]. In the current coronavirus disease 2019 (COVID-19) pandemic, patients presenting with symptoms of respiratory distress along with such suspicious HRCT findings may lead to a critical diagnostic dilemma for physicians, especially with similar clinical and radiological findings. The advent of multiplex diagnostic platforms such as Biofire has proven itself to be an effective diagnostic tool for early and accurate diagnosis, which helps in the proper management of these cases. The RP 2.0 *plus* panel of Biofire covers HCoV-229E, HCoV OC43, HCoV-NL63, HCoV-HKU1, MERS-CoV, adenovirus, human rhino/enterovirus, human metapneumovirus, influenza A and B, parainfluenza 1–4, respiratory syncytial virus, *Bordetella pertussis *, *Bordetella parapertussis *, *Chlamydia pneumoniae* and *Mycoplasma pneumoniae *. Biofire is approved by the the United States Food and Drug Administration (FDA) and has shown better sensitivity than other molecular platforms, viral culture and serological detection methods [18]. It is user-friendly as well as having a very short turnaround time (~70 min), which is much more advantageous than cell culture or any other diagnostic modality. This can indirectly help to avoid unnecessary use of antibiotics and indirectly help to reduce the burden of emerging drug resistance.

HCoV-229E has long been considered to be an agent of the common cold in adult patients and the scarcity of the literature makes it a neglected pathogen. The SARS-CoV-2 pandemic has highlighted the lacunae in respiratory viral diagnostics. HCoV-229E is a challenging pathogen for clinicians and virologists alike. The mystery of its variable presentation, immune evasion and severe manifestations among different patients is yet to be explored.

## Supplementary Data

Supplementary material 1
