# Supplementary material for: The neglected pathogen: case reports of severe lower respiratory tract infection by human coronavirus 229E
Source: Access Microbiol. 2022 Feb 10;4(2):000311. doi: 10.1099/acmi.0.000311 (PMC8941960; doi:10.1099/acmi.0.000311)
Supplement: Supplementary material 1 [file acmi-4-0311-s001.pdf]

Table 1. Clinical characteristics and Outcomes of HCoV229E Cases.

Parameters	**Case 1**	**Case 2**
Presenting complains		
Fever	101°F	102°F
Cough	Yes	Yes
Breathlessness	Yes	Yes
Chronic Liver disease		
Decompensated	Yes	Yes
Comorbidities		
Diabetes Mellitus.	No	No
Hypertension	No	No
COPD	No	No
Thyroid disorders	No	No
Coronary artery disease	No	No
Tuberculosis	No	No
Chest Auscultation findings		
Vesicular breath sound	Reduced	Reduced
Bronchial breath sound	Yes	Yes
Diffuse rales	Yes	Yes
Chest X- ray		
Diffuse opacities	No	Yes
Pleural effusion	Yes	Yes
HRCT-Chest*		

Findings suggestive of viral aetiology	Yes	Yes
ABG analysis		
pH	7.45	7.45
PaO_2 (Partial pressure of O_2)	50.60 mmHg	50.70 mmHg
$PaCO_2$ (Partial pressure of CO_2	32 mmHg	32.3 mmHg
P/F ratio†	240.95	241.40
Haematological Findings		
Hb (Haemoglobin)	9 gm/dl	8.60 gm/dl
TLC (Total Leucocyte Count)	11.10 x 10^9/l	7.90 x 10^9/l
Platelet	153 x 10^9/l	105 x 10^9/l
DLC (Differential Leucocyte Count) (%)	N- 85.40, L- 6.80, M-7.10, E-0.20	N-65.50, L-14.90, M-17.40, E-1.80
LFT (Liver Function Test)		
Bilirubin	6.19 mg/dl	11.21 mg/dl
AST (Aspartate Aminotransferase)	60.10 U/l	53 U/l
ALT (Alanine Aminotransferase)	29.40 U/l	25 U/l
KFT (Kidney Function Test)		
Urea	77.80 mg/dl	24.10 mg/dl
Creatinine	1.29 mg/dl	0.45 mg/dl
Inflammatory Markers		
CRP	56 mg/L	45 mg/L
Procalcitonin	0.12 ng/ml	0.09 ng/ml
Microbiological work-up		

SARS COVID-2 RT PCR	Non-Reactive	Non-Reactive
Anti SARS COVID-2 IgG	Non-Reactive	Non-Reactive
Bacterial multiplex PCR	Negative	Negative
Viral multiplex PCR	HCoV-229E	HCoV-229E
Galactomannan assay	Negative	Negative
Ziehl-Neelsen staining	Negative	Negative
Clinical management‡		
Prophylactic antibiotic use	Yes	Yes
Antiviral (Temiflu)	No	No
Steroid use	Yes	Yes
Supplementary Oxygen use	Yes	Yes
Outcome	Recovered	Recovered

*: HRCT- Chest findings – **Case 1** - multiple discrete rounded nodules with areas of confluence in bilateral upper and right middle lobes along with right-sided pleural effusion **Case 2** - multiple small ill-defined nodules in bilateral lung fields suggestive of infective aetiology along with ground-glass opacity in both lung fields and septal thickening in bilateral lower lobes

†: P/F ratio - arterial pO2 (“P”) from the ABG divided by the FIO2 (“F”) – the fraction (percentage) of inspired oxygen that the patient is receiving.

N-Neutrophil, L-Lymphocyte, M-Monocyte, E-Eosinophil, B-Basophil
